# Hypermethylation and Post-Transcriptional Regulation of DNA Methyltransferases in the Ovarian Carcinomas of the Laying Hen

**DOI:** 10.1371/journal.pone.0061658

**Published:** 2013-04-17

**Authors:** Jin-Young Lee, Wooyoung Jeong, Whasun Lim, Chul-Hong Lim, Seung-Min Bae, Jinyoung Kim, Fuller W. Bazer, Gwonhwa Song

**Affiliations:** 1 WCU Biomodulation Major, Department of Agricultural Biotechnology, Seoul National University, Seoul, Republic of Korea; 2 Department of Animal Resources Science, Dankook University, Cheonan, Republic of Korea; 3 Center for Animal Biotechnology and Genomics and Department of Animal Science, Texas A&M University, College Station, Texas, United States of America; Clermont Université, France

## Abstract

DNA methyltransferases (DNMTs) are key regulators of DNA methylation and have crucial roles in carcinogenesis, embryogenesis and epigenetic modification. In general, DNMT1 has enzymatic activity affecting maintenance of DNA methylation, whereas DNMT3A and DNMT3B are involved in *de novo* methylation events. Although *DNMT* genes are well known in mammals including humans and mice, they are not well studied in avian species, especially the laying hen which is recognized as an excellent animal model for research on human ovarian carcinogenesis. Results of the present study demonstrated that expression of *DNMT1*, *DNMT3A* and *DNMT3B* genes was significantly increased, particularly in the glandular epithelia (GE) of cancerous ovaries, but not normal ovaries. Consistent with this result, immunoreactive 5-methylcytosine protein was predominantly abundant in nuclei of stromal and GE cells of cancerous ovaries, but it was also found that, to a lesser extent, in nuclei of stromal cells of normal ovaries. Methylation-specific PCR analysis detected hypermethylation of the promoter regions of the tumor suppressor genes in the initiation and development of chicken ovarian cancer. Further, several microRNAs, specifically *miR-1741, miR-16c,* and *miR-222,* and *miR-1632* were discovered to influence expression of *DNMT3A* and *DNMT3B*, respectively, via their 3′-UTR which suggests post-transcriptional regulation of their expression in laying hens. Collectively, results of the present study demonstrated increased expression of *DNMT* genes in cancerous ovaries of laying hens and post-transcriptional regulation of those genes by specific microRNAs, as well as control of hypermethylation of the promoters of tumor suppressor genes.

## Introduction

Ovarian cancer is the most common malignancy in the female genital tract in the United States, and the fifth leading cause of cancer-related deaths among women. Of these, the surface epithelial-derived ovarian cancer accounts for 90% of all ovarian cancers [1]. Since the idea that the repeated rupture of the ovarian epithelium during the monthly ovulation event in women may contribute to accelerate the incidence of the epithelial ovarian cancer was coined by Fathalla about 40 years ago [2], the etiology of ovarian cancer is complicated and not fully understood. However, results of a number of epidemiological studies indicate that there is an increased ovarian cancer risk dependent on ovulation frequency and reproductive factors [3]. Recently, the early diagnosis of epithelial ovarian cancer and prediction of prognosis for patient survival using specific biomarkers is increasingly recognized as a better approach to identify this disease. To overcome these limitations and obstacles and to elucidate the etiology and pathogenesis of epithelial ovarian cancer, various genetically engineered rodent models have been developed and they are very useful; however, the artificial nature of the induced tumors in rodents limits their clinical relevance [4], [5], [6]. Meanwhile, the laying hen is the only established animal model that spontaneously develops ovarian surface epithelium-derived tumors. In addition, cysts generation and epithelial dysplasia of the surface epithelium of their ovaries is generally believed to be the precursor of the epithelial-derived ovarian cancer associated with number of ovulations as reported for humans [4]. Furthermore, laying hen animal model shares a number of common pathological features and histological subtypes with human ovarian cancer. [4], [5], [7].

In higher organisms, DNA methylation plays pivotal roles in normal growth/development and cellular differentiation and affects a variety of biochemical events such as genomic imprinting and X-chromosome inactivation [8]. In general, DNA methylation involves the addition of a methyl group to the carbon 5 position (5 meC) of the cytosine residue in the pyrimidine ring [9]. Thereby this modification has the specific effect of reducing gene expression and can be inherited by offspring. DNA methylation events in mammalian cells are mainly carried out by two major classes of enzymatic activities; maintenance methylation via DNA methyltransferase 1 (DNMT1) and *de novo* methylation via DNMT3A and DNMT3B. In cancer biology, overexpression of DNMTs is a hallmark of cancer cells such as endometrioid carcinomas and prostate cancer [10], [11], [12] and it is responsible for aberrant promoter hypermethylation of tumor suppressor genes in various human cancer cells [13], [14]. Although expression and functional roles of DNMTs are well studied in mammalian species, including humans and mice, little is known about their expression and epigenetic regulation in avian species, especially laying hens that develop epithelial ovarian cancer spontaneously. Therefore, the objectives of this study with laying hens were to determine: 1) the expression of DNMTs in normal and cancerous ovaries; and 2) whether DNMTs are regulated by post-transcriptional actions of specific microRNAs using a miRNA target validation assay. Our results confirm that the laying hen is an established excellent model for research on human ovarian cancer and that DNMTs may play a key role in ovarian carcinogenesis.

## Results

### Patterns of Expression and Cell-specific Localization of DNMT1, DNMT3A and DNMT3B mRNAs in Normal and Cancerous Ovaries of Laying Hens

**Figure 1:**
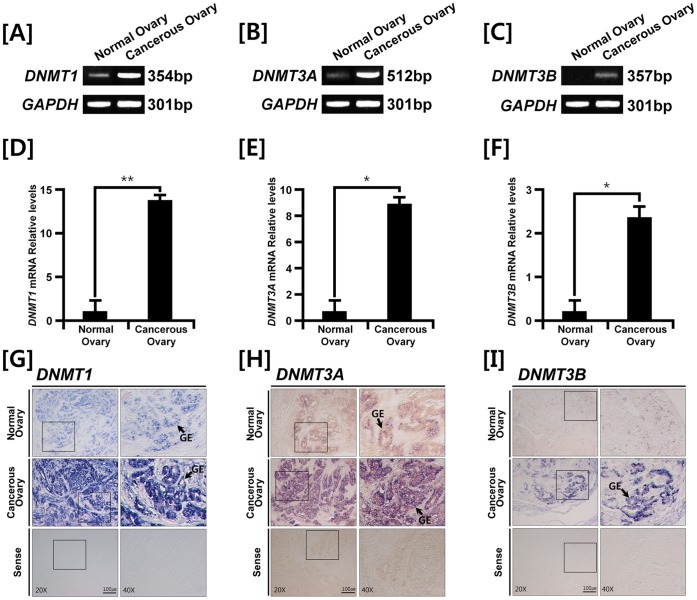
Expression, quantitation and localization of *DNMTs* in normal and cancerous ovaries from laying hens. [A–C] RT-PCR and [D–F] q-PCR analyses were performed using cDNA templates from normal and cancerous ovaries of laying hens using chicken *DNMT1, DNMT3A, DNMT3B* and *GAPDH* primers. The asterisks denote statistically significant differences (**P<0.01 and *P<0.05). [G–I] *In situ* hybridization analyses of *DNMT* mRNAs in normal and cancerous ovaries of hens. Cross-sections of normal and cancerous ovaries of hens hybridized with antisense or sense chicken *DNMT* cRNA probes. Legend: GE, glandular epithelium. See *Materials and Methods* for a complete description of the methods.

To determine if *DNMTs* are up- or down-regulated in ovarian cancer cells of our laying hen model, we performed RT-PCR and quantitative RT-PCR analyses. Results of the present study identified three *DNMT* mRNAs that are unique to ovarian carcinomas in laying hens (Figure 1A to –C). Further, quantitative PCR revealed that expression of *DNMT1, DNMT3A* and *DNMT3B* mRNAs increased 13.8- (*P*<0.01), 9.1- (*P*<0.05), and 2.7-fold (*P*<0.05) in the cancerous ovaries, respectively, as compared with normal ovaries of laying hens (Figure 1D to –F). Further, *in situ* hybridization analysis revealed that the three *DNMT* mRNAs were abundantly expressed in glandular epithelium (GE) of cancerous ovaries, but not in stroma and blood vessels (Figure 1G to –I). Consistent with results of PCR analyses, expression of *DNMT* mRNAs in GE of normal ovaries was extremely weak.

### DNA Methylation Patterns in Normal and Cancerous Ovaries of Hens

**Figure 2:**
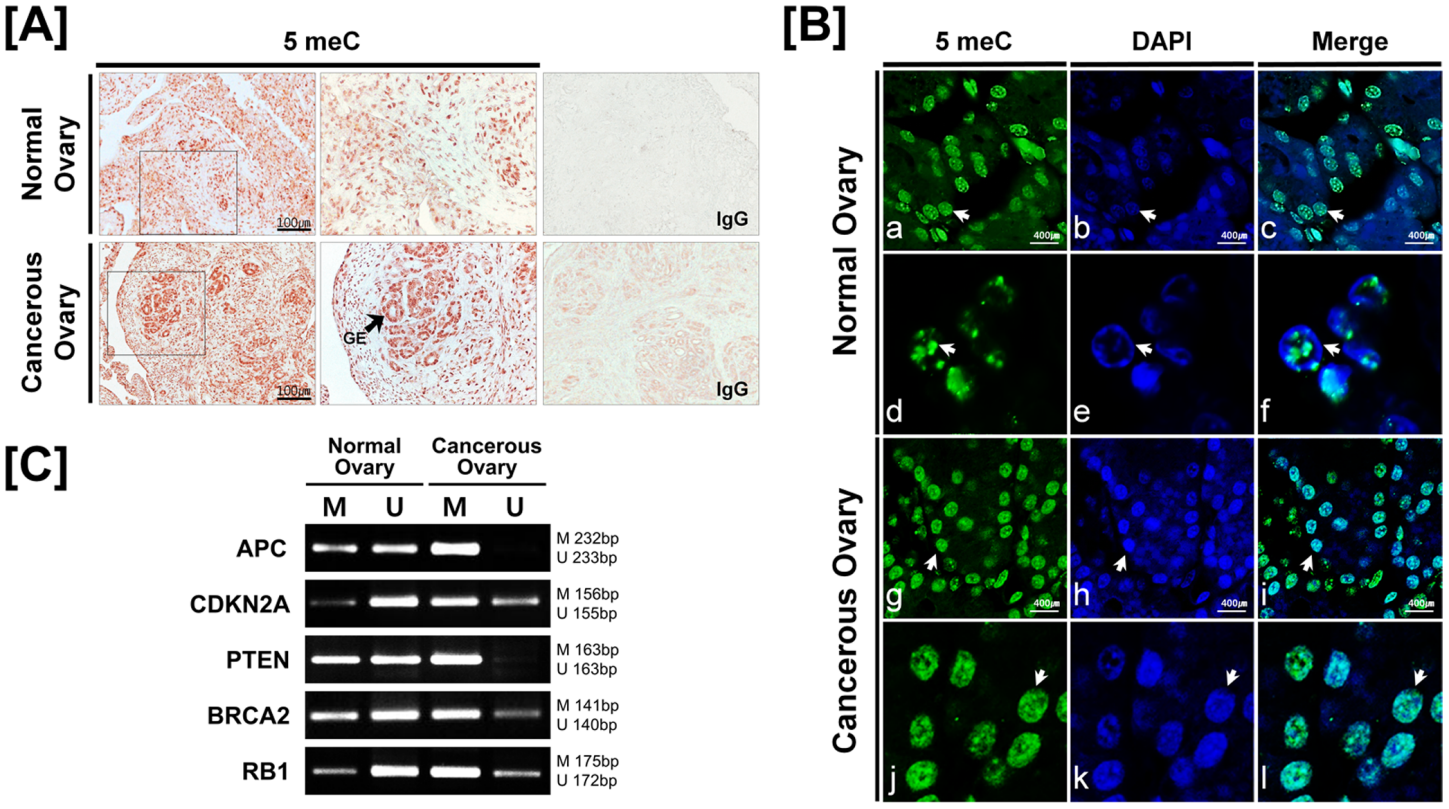
Methylation patterns of DNMTs and tumor suppressor genes in normal and cancerous ovaries from laying hens. [A and B] Localization of 5-methylcytosine protein in normal and cancerous ovaries of hens. Sections were not counterstained. Arrows in panel B indicate nuclei in the glandular epithelium of ovaries. [C] Methylation status of promoter regions of tumor suppressor genes using methylation-specific PCR analyses. Legend: GE, glandular epithelium; M, methyl primer; U, unmethyl primer. See *Materials and Methods* for a complete description of the methods.

To compare general methylation patterns in normal and cancerous ovaries from laying hens, we performed immunohistochemistry analysis using an antibody to 5-methylcytocine (5 meC). As shown in Figure 2A, immunoreactive 5 meC protein was localized in the GE and stromal cells of cancerous ovaries, and also detected at low abundance in the stromal cells of normal ovaries. Similarly, immunofluorescence staining demonstrated that immunoreactive 5 meC protein was predominantly abundant in nuclei of stromal and GE cells of cancerous ovaries, but it was also found that, to a lesser extent, in nuclei of stromal cells of normal ovaries (Figure 2B). This indicates that GE cells in normal ovaries are not undergoing DNA methylation, whereas GE cells in cancerous ovaries have or are undergoing DNA methylation.

### DNA Methylation Pattern of Promoter Regions of DNMTs and Tumor Suppressor Genes

To investigate the DNA methylation status of the promoter regions of selected tumor suppressor genes such as *APC, CDKN2A*, *PTEN, BRCA2,* and *RB1*, we performed methylation-specific PCR analysis. As illustrated in Figure 2C, the unmethylation status of *APC, CDKN2A,* and *RB1* is higher than their methylation status in normal ovaries, whereas those genes are highly methylated in cancerous ovaries. Similarly, the product band intensity of the methylation or unmethylation statuses of the *PTEN* and *BRCA2* promoter regions are equivalent in normal ovaries; but both regions are predominantly methylated in cancerous ovaries.

### Post-transcriptional Regulation of microRNA Affecting DNMTs

**Figure 3:**
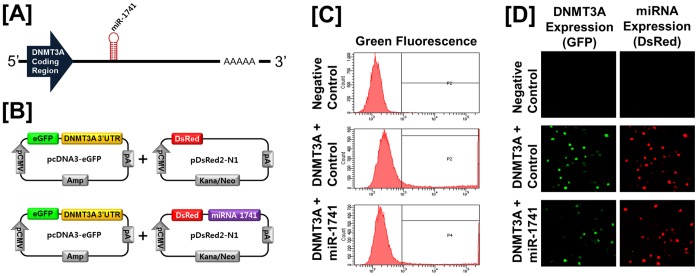
In vitro target assay for microRNAs of the DNMT3A transcript. [A] Diagram of *miR-1741* binding site in the *DNMT3A* 3′-UTR. [B] Expression vector map for eGFP with *DNMT3A* 3′-UTR and Ds-Red with *miR-1741*. [C and D] After co-transfection of pcDNA-eGFP-3′-UTR for the *DNMT3A* transcript and pcDNA-DsRed-miRNA for the *miR-1741*, the fluorescence signals of GFP and DsRed were detected using FACS [C] and fluorescent microscopy [D].

**Figure 4:**
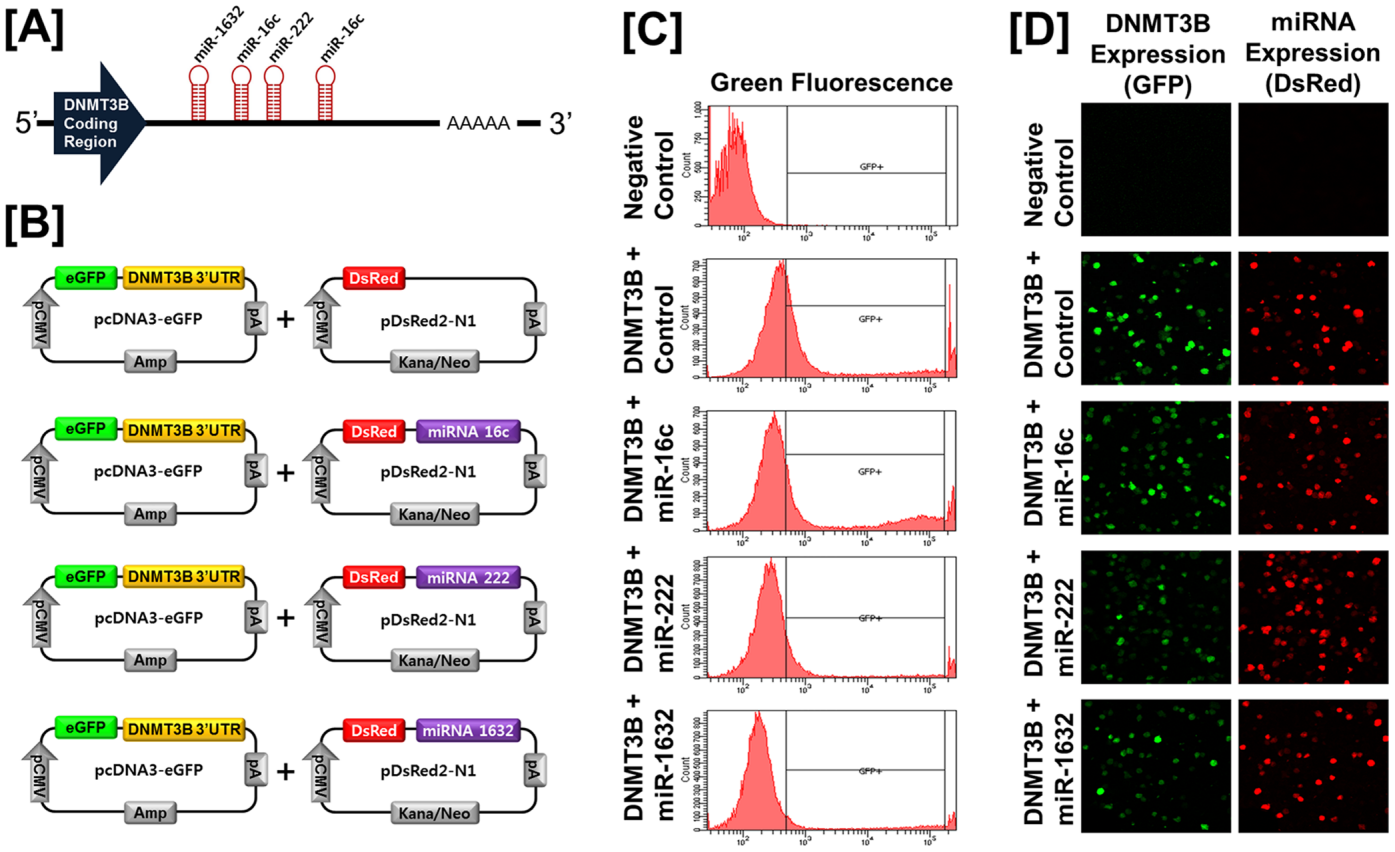
In vitro target assay of microRNAs on the DNMT3B transcript. [A] Diagram of *miR-16c*, *miR-222*, and *miR-1632* binding sites in the *DNMT3B* 3′-UTR. [B] Expression vector map for eGFP with *DNMT3B* 3′-UTR and Ds-Red with each miRNA. [C and D] After co-transfection of pcDNA-eGFP-3′-UTR for the *DNMT3B* transcript and pcDNA-DsRed-miRNA for the *miR-16c*, *miR-222*, and *miR-1632*, the fluorescence signals of GFP and DsRed were detected using FACS [C] and fluorescent microscopy [D].

Based on the possibility that expression of chicken *DNMT* genes is regulated at the post-transcriptional level by microRNAs (miRNAs), we performed a miRNA target validation assay. Analysis of potential miRNA binding sites within the 3′-UTR of the each *DNMT* gene using the miRNA target prediction database (miRDB; http://mirdb.org/miRDB/) revealed putative binding sites for *miR-148a* and *miR-1612* (for *DNMT1*)*; miR-1596, miR-1687, miR-1741,* and *miR-1749* (for *DNMT3A*)*;* and *miR-16c, miR-222,* and *miR-1632* (for *DNMT3B*). Therefore, we determined whether these miRNAs influenced expressions of each *DNMT* gene via its 3′-UTR. A fragment of the 3′-UTR of each gene harboring binding sites for the miRNAs were cloned in downstream of the green fluorescent protein (GFP) reading frame, thereby creating a fluorescent reporter for function of the 3′-UTR region. After co-transfection of eGFP-3′-UTR of each gene and DsRed-miRNA, the intensity of GFP expression and percentage of GFP-expressing cells were analyzed by fluorescence microscopy and fluorescence activated cell sorting (FACS). As illustrated in Figure 3, in the presence of *miR-1741* for *DNMT3A*, the intensity and percentage of GFP-expressing cells (100% in control vs. 73.15% in *miR-1741*) decreased (*P*<0.01). However, in the presence of *miR-1596, miR-1687,* or *miR-1749*, neither the intensity nor percentage of GFP-expressing cells changed (data now shown). In addition, as shown in Figure 4, in the presence of *miR-16c, miR-222,* or *miR-1632* for *DNMT3B*, there was a decrease (P<0.01) in the percentage of GFP-expressing cells (100% in control vs. 85.3% in *miR-16c*, 40.3% in *miR-222*, and 25.9% in *miR-1632*). In the presence of *miR-148a* or *miR-1612* for *DNMT1,* neither the intensity nor percentage of GFP-expressing cells changed (data now shown). These results indicate that *miR-1741, miR-16c, miR-222,* or *miR-1632* directly bind to *DNMT3A* or *DNMT3B* transcripts, respectively, and post-transcriptionally regulate expression of the *DNMT3A* and *DNMT3B* genes.

## Discussion

Key findings of the present study were that expression of the *DNMT1, DNMT3A* and *DNMT3B* genes are abundantly expressed only in GE of cancerous ovaries as compared to normal ovaries of laying hens, and that expression of *DNMT3A* and *DNMT3B* genes are post-transcriptionally regulated by *miR-1741, miR-16c, miR-222,* or *miR-1632*, respectively. These results support our hypothesis that DNMTs are critical regulators of initiation, growth and development of epithelial-derived ovarian cancer in hens. Generally, tumorigenesis is associated with accumulation of genetic changes such as mutation, rearrangement, deletion and translocations in genes. However, these classical theories alone were unable to clarify the basis for carcinogenesis, and it is now understood that epigenetic events involving multiple interactions with DNMTs, small non-cording RNAs and tumor suppressor genes likely lead to ovarian carcinogenesis. In the present study, we mainly focused on multiple epigenetic mechanisms involved in the regulation of *DNMT* genes in normal and cancerous ovaries of laying hens, which are the most relevant animal model to identify biomarkers of human ovarian cancer such as CA125, cytokeratin, EGFR, Lewis Y, and erbB-2 and also expressed in carcinoma cells, but not normal cells in ovaries of laying hens [15], [16], [17], [18].

As a major epigenetic modification, DNA methylation affects various biochemical processes such as regulation of gene transcription, maintenance of genomic stability and imprinting, and X-chromosome inactivation in mammals [19]. In fact, all DNMTs have functional roles in regulation of DNA methylation. DNMT1, as a member of the maintenance-type methyltransferase family, consists of an N-terminal regulatory domain, glycine-lysine repeat and C-terminal catalytic domains and is predominantly responsible for hemimethylated CpG di-nucleotides in the mammalian genome [20]. Indeed, appropriate expression of DNMT1 is essential for the preservation of parental imprinting [21]. For instance, in mice, although *Dnmt1^−/−^* embryonic stem cells are viable, have no obvious abnormalities related to growth rate or morphology and contain a small percentage of methylated DNA and methyltransferase activity, the *Dnmt1^−/−^* embryos are stunted in development and die during mid-gestation [22]. Furthermore, overexpression of DNMT1 is a hallmark of endometrioid carcinomas and prostate cancer [10] and it is also responsible for both *de novo* and maintenance methylation of tumor suppressor genes in various human cancer cells [13]. On the other hand, DNMT3A and DNMT3B could methylate hemimethylated or unmethylated CpG islands at the same rate. Although the general architecture of DNMT3 enzymes is very similar to that of DNMT1, their total length is shorter than DNMT1 and they have an unique tetrapeptide of proline-tryptophan-tryptophan-proline (PWWP) motif [23]. Likewise *Dnmt1^−/−^*, *Dnmt3a^−/−^* and *Dnmt3b^−/−^* mice experience embryonic lethality during gestation or early in the neonatal period due to hypomethylation of pericentrimeric repeats [24]. In addition, overexpression of either DNMT3A or DNMT3B is associated with tumorigenesis depending on cancer types in humans [11], [12]. These results indicate that both DNMT3A and DNMT3B function as *de novo* methyltransferases that play important roles in normal development and disease.

Consistent with previous reports, results of the present study demonstrate that expression levels of *DNMT1, DNMT3A* and *DNMT3B* genes are significantly increased in cancerous as compared with normal ovaries (Figure 1). Furthermore, all *DNMT* mRNAs were predominantly abundant in GE of cancerous ovaries. In fact, a number of complex glandular architectures are usually found in various carcinomas that arise in various organs such as stomach, bronchus, bladder, prostate, testis and ovary due to the ubiquitous nature of glands. This is especially true for ovaries of both avian and mammalian species, as these glandular structures are mainly detected in the endometrioid-type tumors with characteristics such as nuclear atypia, cribriform foci and atresia of stromal follicles [4]. In addition, as illustrated in Figures 2A and 2B, immunoreactive 5-methylcytosine protein was predominantly abundant in the GE cells of cancerous ovaries which indicate that these GE cells are undergoing DNA methylation in response to increased expression of DNMTs. Further, methylation-specific PCR data demonstrated that there was a significant increase in methylation patterns of the promoter regions of *APC, CDKN2A, PTEN, BRCA2,* and *RB1* which are tumor suppressor genes. These results support the idea that epigenetic silencing of tumor suppressor genes by promoter CpG island hypermethylation is one of the most important regulatory mechanisms leading to the generation and proliferation of carcinomas [25]. Recently, Socha and colleagues reported that secreted protein acidic and rich in cysteine (SPARC) is down-regulated in ovarian cancer through aberrant promoter hypermethylation [12]. Additionally, recent results demonstrated under-expression of tumor suppressor genes in response to hypermethylation on their promoter regions in various tumor types, such as bladder, gastric and gynecological cancers [26], [27], [28]. Indeed, global DNA hypomethylation and locus- and gene-specific DNA hypermethylation have been implicated as hallmarks of many cancers [29]. Likewise, results of the present study indicate that silencing of *APC, CDKN2A, PTEN, BRCA2* and *RB1* genes by promoter hypermethylation occurs in ovarian tumors, suggesting the importance of changes in methylation patterns on the promoter regions of these tumor suppressor genes in ovarian carcinogenesis.

MicroRNAs (miRNAs) are small and non-coding RNAs of 18–23 nucleotides in length that regulate gene expression post-transcriptionally and alter cell fate by controlling translation of target mRNAs in diverse tissues and cell types. Thus, miRNAs play crucial roles in various biological processes including vertebrate growth, development, differentiation and oncogenesis by regulating gene expression [30]. In the present study, our miRNA target validation assay demonstrated that in the presence of *miR-1741* for *DNMT3A*, the intensity and percentage of GFP-expressing cells decreased (*P*<0.01), but this did not occur in the presence of *miR-1596, miR-1687,* or *miR-1749*. Similarly, the presence of *miR-16c, miR-222,* or *miR-1632* for *DNMT3B*, the percentage of GFP-expressing cells was decreased (*P*<0.01). These results indicate that *miR-1741, miR-16c, miR-222,* or *miR-1632* directly bind to the *DNMT3A* or *DNMT3B* transcript, respectively, and post-transcriptionally regulate expression of those genes.

Collectively, results of the present study are the first to demonstrate distinct cell-specific expression patterns for *DNMT*s genes and determine the methylation status of CpG islands of promoter regions of tumor suppressor genes between normal and cancerous ovaries of laying hens. Further, our results revealed that *DNMT* gene expression is post-transcriptionally regulated by several miRNAs critical to ovarian carcinogenesis of laying hens. DNA methylation is required for normal embryonic development, X-chromosome inactivation and gene imprinting in mammalian species and its aberrant effects leading to promoter hypermethylation of tumor suppressor genes by inappropriate expression of DNMTs contributes to development of ovarian cancer. Therefore, results of the present study provide new insights into DNMTs with respect to epigenetic regulation and functional roles in ovarian carcinogenesis in laying hens that are likely highly relevant to the development of therapies for treatment of ovarian cancers in humans.

## Materials and Methods

### Experimental Animals and Animal Care

The experimental use of chickens for this study was approved by the Institute of Laboratory Animal Resources, Seoul National University (SNU-070823-5). All White Leghorn (WL) chickens were exposed to a light regimen of 15 h light and 9 h dark, *ad libitum* access to feed and water, and standard management practices for laying hens.

### Tissue Samples

A total 136 laying hens (88 over 36 months and 48 over 24 months of age), which had completely stopped laying eggs were euthanized for biopsy and cancerous (n = 10) ovaries were collected. As a control, normal (n = 5) ovaries were also collected from egg-laying hens. We examined the tumor stage in 10 hens with cancerous ovaries using characteristic features of ovarian cancer, based on the cellular subtypes and patterns of cellular differentiation with reference to malignant tumor types in human ovaries [4], [31]. Three hens had stage III disease as ovarian tumor cells had metastasized to the gastrointestinal (GI) tract and liver surface with profuse ascites in the abdominal cavity. Five hens had tumor cells spread to distant organs including liver parenchyma, lung, GI tract and oviduct with profuse ascites, indicating stage IV disease. Two hens had stage I disease as tumors were limited to their ovaries.

### RNA Isolation

Total cellular RNA was isolated from frozen tissues using Trizol reagent (Invitrogen, Carlsbad, CA) according to manufacturer’s recommendations. The quantity and quality of total RNA was determined by spectrometry and denaturing agarose gel electrophoresis, respectively.

### Semi-quantitative RT-PCR Analysis

The level of expression of *DNMT* mRNAs in normal and cancerous ovaries from chickens was assessed using semi-quantitative as described previously [32]. Complementary DNA (cDNA) was synthesized from total cellular RNA (2 ug) using random hexamer (Invitrogen, Carlsbad, CA) and oligo (dT) primers and AccuPower® RT PreMix (Bioneer, Daejeon, Korea). The cDNA was diluted (1∶10) in sterile water before use in PCR. After PCR, equal amounts of reaction product were analyzed using a 1% agarose gel, and PCR products were visualized using ethidium bromide staining.

### Quantitative RT-PCR Analysis

Gene expression levels were measured using SYBR® Green (Sigma, St. Louis, MO, USA) and a StepOnePlus™ Real-Time PCR System (Applied Biosystems, Foster City, CA, USA) [33]. The *GAPDH* gene was simultaneously analyzed as a control and used for normalization to account for variation in loading. Each target gene and *GAPDH* was analyzed in triplicate. ROX dye (Invitrogen) was used as a negative control for the fluorescence measurements. Sequence-specific products were identified by generating a melting curve in which the C_T_ value represented the cycle number at which a fluorescent signal was statistically greater than background, and relative gene expression was quantified using the 2^–ΔΔCT^ method [34]. For the control, the relative quantification of gene expression was normalized to the C_T_ of the control ovary.

### In Situ Hybridization Analysis

Location of mRNA in sections (5 µm) of chicken oviduct and ovaries was determined by non-radioactive *in situ* hybridization analysis as described previously [31]. After verification of the sequences, plasmids containing the correct gene sequences were amplified with T7- and SP6-specific primers and then digoxigenin (DIG)-labeled RNA probes were transcribed using a DIG RNA labeling kit (Roche Applied Science, Indianapolis, IN). After hybridization and blocking, the sections were incubated overnight with sheep anti-DIG antibody conjugated to alkaline phosphatase (Roche). The signal was visualized by exposure to a solution containing 0.4 mM 5-bromo-4-chloro-3-indolyl phosphate, 0.4 mM nitroblue tetrazolium, and 2 mM levamisole (Sigma).

### Immunohistochemistry

Immunocytochemical localization of 5-methylacytosine protein in normal and cancerous ovaries from chickens was performed using a mouse monoclonal antibody to 5-methylcytosine (catalog number ab-10805; AbCam, CA, USA) at a final dilution of 1∶200 (0.2 µg/ml) as described previously [33]. Negative controls included substitution of the primary antibody with purified non-immune mouse IgG at the same final concentration.

### Immunofluorescence

Immunocytochemical localization of 5-methylacytosine protein in normal and cancerous ovaries from chickens was performed using a mouse monoclonal antibody to 5-methylcytosine (catalog number ab-10805; Abcam, CA, USA) at a final dilution of 1∶200 (0.2 µg/ml) as described previously [31].

### Methylation-specific PCR (MSP) Analysis

To investigate differential methylation patterns of selected tumor suppressor genes including APC gene (*APC*), *cyclin-dependent kinase inhibitor 2A* (*CDKN2A*, also known as *p16*), *phosphatase and tensin homolog* (*PTEN*), brac2 gene (*BRCA2*), and rb1 gene (*RB1*) between normal and cancerous ovaries, we performed methylation-specific PCR analysis. DNA samples were prepared using an AccuPrep Genomic DNA Extraction Kit (Bioneer) and converted using Epitect Bisulfite kit (QIAGEN, Doncaster, Australia) according to the manufacturer’s instructions. PCR analyses were performed with both a methylation-specific primer and an unmethylation-specific primer for each gene with forward and reverse primers.

### MicroRNA Target Validation Assay

The 3′-UTRs of *DNMTs* were cloned and confirmed by sequencing. Each 3′-UTR was subcloned between the *eGFP* gene and the bovine growth hormone poly-A tail in pcDNA3eGFP (Clontech, Mountain View, CA) to generate the eGFP-miRNA target 3′-UTR (pcDNA-eGFP-3′UTR) fusion constructs as described previously [32]. For the dual fluorescence reporter assay, the fusion contained the *DsRed* gene and either *miR-148a* or *miR-1612* for *DNMT1; miR-1596, miR-1687, miR-1741,* or *miR-1749* for *DNMT3A;* and *miR-16c, miR-222,* or *miR-1632* for *DNMT3B*, and each was designed to be co-expressed under control of the CMV promoter (pcDNA-DsRed-miRNA). At 48 h post-transfection, dual fluorescence was detected by fluorescence microscopy and calculated by FACSCalibur flow cytometry (BD Biosciences). For flow cytometry, the cells were fixed in 4% paraformaldehyde and analyzed using FlowJo software (Tree Star Inc., Ashland, OR).

### Statistical Analyses

Data obtained using quantitative PCR analysis are presented as mean ± SEM unless otherwise stated. Differences in the variances between normal and cancerous ovaries were analyzed using the *F* test, and differences between means were subjected to the Student’s *t* test. Differences with a probability value of *P*<0.05 were considered statistically significant.
